# Preliminary results of adapting the stepped care model for depression management in Vietnam

**DOI:** 10.3389/fpsyt.2022.922911

**Published:** 2022-08-18

**Authors:** Mai Tuyet Do, Tam Thanh Nguyen, Huong Thi Thanh Tran

**Affiliations:** ^1^Hanoi Medical University, Hanoi, Vietnam; ^2^BasicNeeds Vietnam, Hanoi, Vietnam; ^3^Vietnam National Cancer Institute, Hanoi, Vietnam

**Keywords:** adapting, stepped care model, depression, management, Vietnam, community

## Abstract

**Background:**

Depression is the leading burden of mental disease, especially in low-and-middle-income countries like Vietnam. The Stepped Care Model is a promising approach to managing depression in the community with low resources. This is the first study that implemented the adapted Stepped Care Model for depression management in the Vietnamese context and evaluated the initial effectiveness of this community-based intervention in the Thai Nguyen community.

**Materials and methods:**

A quasi-experimental study with a 3-month follow-up was conducted in 10 selected communes in Thai Nguyen province. The most important modifications in the Stepped Care Model for depression management were the screening focused on the high-risk individuals living in the community; the combination of 8-session group psychotherapy with animation activities at commune health stations (CHS); and using psychotherapy as first-line treatment. From August 2020 to January 2021, quantitative data were collected using the Patient Health Questionnaire-9, the Generalized Anxiety Disorder-7, and the Quality of Life Enjoyment and Satisfaction Questionnaire-Short Form. The descriptive analyses were performed to describe the demographic characteristics and the change in the questionnaires' mean score at the baseline and 3-month follow-up.

**Results:**

A total of 1,891 people were approached in the community, of which 359 voluntary people met the study criteria and attended group psychotherapy. During group psychotherapy, the average PHQ-9 scores gradually decreased, and after the 8th session, this figure dropped by 2.65 times compared to the beginning. After 3 months, the percentage of the group with mild-moderate depression shrank from 95.5 to 9.3%, and there were no more severe cases. Moreover, life satisfaction increased by 32% and the anxiety level significantly dropped according to Q-LES-Q-SF and GAD-7 accordingly.

**Conclusion:**

The preliminary results after 3-month follow-up showed that the Stepped Care Model with group psychotherapy at the CHS was promising to manage the depression in the community. This task shifting approach with limited resources should be further disseminated and studied for long-term effectiveness in low-and-middle-income countries like Vietnam.

## Introduction

Depression is one of the most prevalent mental disorders that presents with depressed mood, loss of interest or pleasure, and reduced energy (1). The World Health Organization (WHO) estimates that the number of people with depressive symptoms is over 350 million internationally, predicting the third leading cause of the global burden of disease by 2030 and leading to nearly 800,000 suicidal deaths each year (2). In low-to-middle-income countries (LMIC), the treatment gap for depression is large, with the majority of those in need receiving little or no treatment (3). Antidepressants and psychological interventions for depression exist (4, 5), and have been shown to be effective in a variety of different countries (6) including Vietnam (7). In LMICs, significant barriers that contribute to this treatment gap are the lack of human resources in mental health care, significant stigma, and limited knowledge about depression (8).

To address this gap, task-shifting and integration of mental health services into other priority health sectors has been recommended to increase access and availability of depression care (9–11). According to WHO, depression is a disorder that can be reliably diagnosed and treated in primary care (12). The Partner in Care program in the United States and the MANAS program developed in India proved that the collaborative task-shifting approaches can mobilize not only the existing health care system but also social support networks. Based on these models, the Stepped Care Model, defined when all patients start with evidence-based treatment of low intensity and only patients who require further treatment step up to a treatment of higher intensity, is proved to be a promising and effective approach to manage the depression in the community, especially in LMICs such as Vietnam (11, 13). A systematic review and meta-analysis of the Stepped care treatments for depression has shown a moderate effect with significant effect size of *d* = 0.34 (95% confidence interval 0.20 to 0.48) (14). The principle of stepped care has been endorsed by many guidelines of depression treatment such as National Institute for Health and Clinical Excellence (15, 16) and Dutch multidisciplinary guideline (17).

In Vietnam, the Stepped Care Model was adapted to Collaborative Stepped Care Model for Depression in which depression care was task-shifted from specialized care to primary care system, and specialized health staff worked collaboratively with general health and non-health workers. This program combined a training model of psychosocial intervention skills for commune health station (CHS) staff to screen for depression, provide psychoeducation, guideline medications, and/or individual behavior activation therapy under the supervision of provincial psychiatrists (7, 18). However, the implementation and effectiveness of the Stepped Care Model for depression with group-based psychotherapy in the Vietnamese context have not been implemented and evaluated yet. Therefore, this study was conducted to (1) investigate the depression situation in Thai Nguyen province in 2020, and (2) evaluate the initial effectiveness of community-based intervention with the Stepped Care Model for depression in Thai Nguyen community.

## Materials and methods

### The adapted collaborative stepped care model for depression in Vietnam

**Figure 1:**
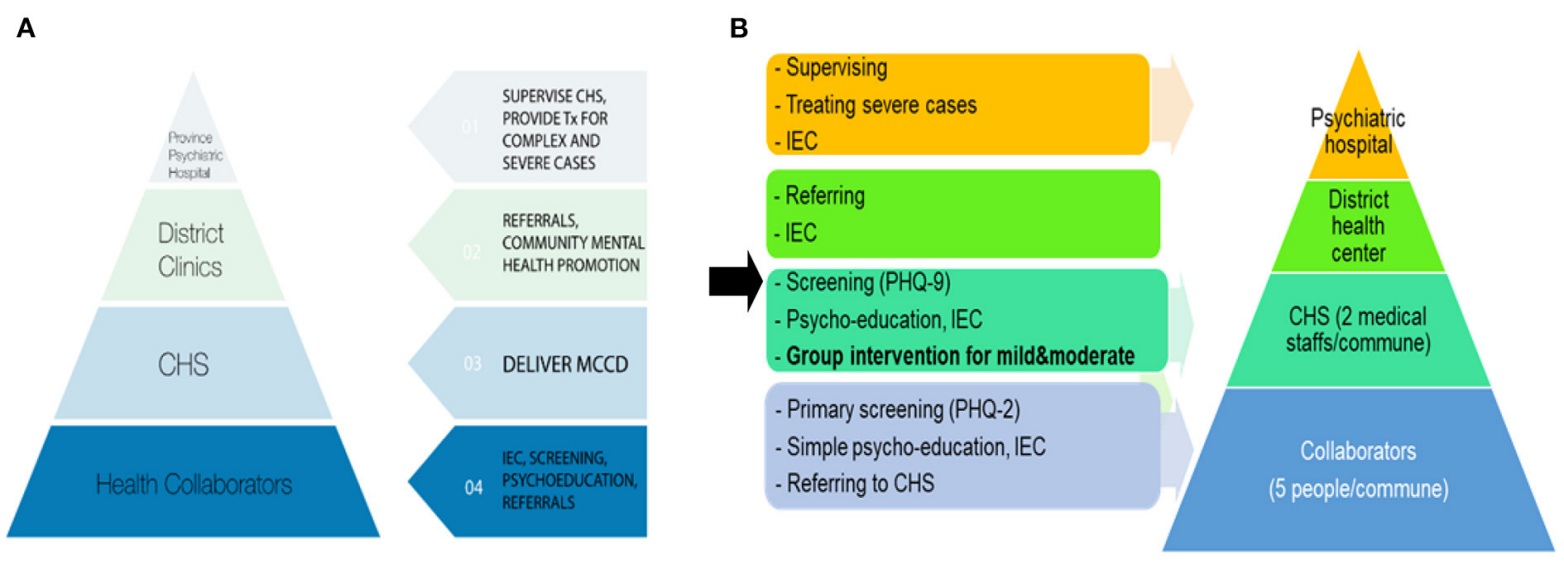
The adapted Stepped Care Model in Vietnam from 2009 to 2020. MCCD, Model of Collaborative Stepped-Care for Depression; Tx, treatment; IEC, Information, Education, Communication; CHS, Commune Health Station. **(A)** The first Stepped Care Model for people with depression was implemented in Vietnam community in 2009 with the name “Model of Collaborative Stepped-Care for Depression.” This model consisted of 4 steps: from primary screening by local health collaborators in the community with the PHQ-2; to secondary screening by CHS staff with the PHQ-9 under the supervision of psychiatrists at CHS; then referring to district health clinics or provincial psychiatric hospital for advanced treatment if necessary (medication or intensive psychotherapy). The task-shifting intervention at the CHS include psychoeducation, guideline antidepressant medications, and/or 5-session individual behavioral activation therapy with the support of mental health specialists. **(B)** The adapted Stepped Care Model for people with depressive symptoms was implemented in Vietnam community in 2020 with the same four steps, three main modifications including primary screening focus on the high-risk individuals (e.g., poor people, people suffering from household violence or chronic health problems); trained CHS staff provided group-based psychotherapy focusing on behavioral activation, problem-solving and effective communication skills; and using psychotherapy as first-line treatment without medication.

Vietnam currently is reported to be the fifteenth-most populous country in the world with a population of more than 98 million people (*Vietnam Population (20) - Worldometer*). The annual gross domestic product per capita in 2020 is $2.785, doubling the figure of 2010, which has resulted in a change from poor to LMIC status (19). Together with the rapid economic growth, the social consequences are becoming apparent, leading to increased risk factors of depression. Due to the lack of resources and severe stigma, mental health services continue to lag far behind the need and largely focus on just medication and severe mental illness in limited institutional settings. To deal with these barriers, the Stepped Care approach was first piloted in Vietnam in 2009 with a collaborative stepped care model, (Figure 1) based on the Partners in Care collaborative care program for depression developed in the United States and the MANAS program developed in India (11, 20). This model used a task-shifting approach in which non-specialized personnel was trained to deliver low-cost depression care including psychoeducation, guideline antidepressant medications, and/or 5-session individual behavioral activation therapy with the support of mental health specialists. It has been found to be a suitable and effective model to manage depression in the Vietnamese community (7).

In this study, the Stepped Care Model is further adapted to identify and manage mild to moderate depression cases in the community. The most important changes were the screening focus on the high-risk individuals (e.g., poor people, people suffering from household violence or chronic health problems); trained CHS staff provided group-based psychotherapy focusing on behavioral activation, problem-solving and effective communication skills; and using psychotherapy as first-line treatment. The implementation of the modified Stepped Care Model consists of five steps: (1) Training for healthcare staffs and collaborators on awareness-raising, mental health promotion, screening for depression, psychoeducation, group-based behavioral activation therapy, referral, and supportive supervision skills; (2) Screening for depression with the PHQ-2 (PHQ-2 score ≥2) and referral in the community; (3) Screening for depression with the PHQ-9 (PHQ-9 score ≥10) and referral in the CHS if needed (high risk of severe mental disorders); (4) Group-based psychotherapy intervention at the CHS by trained CHS staff under the supportive supervision of provincial psychiatrists, 90 minutes each week; and (5) Follow up after 3 months. Specifically, the contents of 8-session group intervention were: (1) Introduction and motivation, (2) Doing beneficial activities for health, (3) Choosing appropriate activities and keeping life balanced, (4) Addressing motivation and plans, (5) Problem-solving skills, (6) Importance of social network, (7) Simple and effective communication skills, and (8) Relapse prevention and graduation.

### Study design

We used a quasi-experimental research design that included a single-arm, pre- and post-intervention assessment of outcomes. Symptoms of depression (the PHQ-9 score) were measured before the intervention (“baseline”); at the week 2, 4, 6 and at the end of the 8-week intervention; and at 3 months post-intervention. Symptoms of anxiety (GAD-7 score) and quality of life (Q-LES-Q-SF score) were measured at baseline; and 3 months post-intervention.

### Study site and time

The research is implemented from August 2020 to January 2021 at Thai Nguyen province, which is a northern mountainous midland province of Vietnam with a population of more than 1.2 million and a poverty rate of 9.2% (21). This study targeted the people living in the community in 10 selected communes from one district of Thai Nguyen province including Quyet Thang, Tan Thinh, Thinh Dan, Tan Lap, Trung Thanh, Gia Sang, Huong Son, Cam Gia, Tich Luong, Phu Xa.

### Sample size and sampling

The sample size of group intervention was calculated using the formula for estimating the sample size in the before-after study: *n* = (2C (1-r))/(ES)2, in which C = 13 with α = 0.05 and β = 0.05; *r* = 0.6; ES = d / s with d = 2, and s = 6.12 that is the standard deviation of the PHQ-9 score in the depressed patient group in Vietnam (21). Thus, the minimum sample size of the intervention group (*n*) is 97. According to Tran Viet Nghi (22), the rate of depression in one commune of Thai Nguyen province was 8.35%, so that the minimum pre-screening PHQ-2 population in our study is *N* = 97 x 8,35 = 809.95. Therefore, the study was expected to screen the depression in at least 891 people by using PHQ-2 in the community. In fact, our study screened for 1,891 people in the community.

The sampling was conducted with the inclusion criteria including (1) age above 18, (2) settling in selected communes, (3) voluntarily accepting to participate in the study; and the exclusion criteria was having serious physical or mental disorders that could interfere with the interaction.

### Procedure of study

The volunteer village health workers were trained about awareness-raising, mental health promotion, and screening for depression with the PHQ-2 (Patient Health Questionnaire, two items). These local health collaborators with a deep understanding of the community and the circumstances of local families approached high-risk people at home (i.e., poor people, people suffering from household violence or chronic health problems, caregivers of people with chronic severe diseases) and screened for depression with the PHQ-2. All people with PHQ-2 score ≥2 were referred to CHS for clinical screening with PHQ-9 (Patient Health Questionnaire, nine items) by medical staffs and diagnosing by psychiatrists. The specialists screened the depressive participants for the risk of severe mental disorders, and, if any, referring to the provincial psychiatric hospital for higher level treatment. All the people with PHQ-9 score ≥10 were provided the simple psycho-education about the depression and information about the study, and then were invited to participate. After that, the voluntary participants were asked to provide their informed consents before joining the group. The group intervention consists of weekly 8-session group-based behavioral activation therapy with animation activities at the CHS with the supervision of local psychiatrists. Each group involved 14–20 people with depressive symptoms (PHQ-9 score ≥10) joined during about 90 min with the facilitation of 2 CHS staff. The intervened participants were interviewed at baseline and after 3 months follow-up with a standard set of questionnaires.

### Study measurements and variables

The study used a paper set of questionnaires including the general information and the psychometric tests. The general information consists of age, gender, educational level, and marital status. The depression symptoms were evaluated by the PHQ-2 in the community, and the PHQ-9 at the CHS.

The PHQ-9 including nine questions was developed by Kroenke et al. in 2001 based on the diagnostic criteria of depression in the Diagnostic and Statistical Manual of Mental Disorders, 4th edition (DSM-IV) (23). For each item, the response options can be “not at all,” “several days,” “more than half the days,” and “nearly everyday,” scored as 0, 1, 2 and 3, respectively. A score of 0–4 suggests no depression; scores of 5–9 shows mild depression; a score of 10–14 represent moderate depression; and a score ≥15 indicates severe depression. This screener has been validated among Vietnamese populations (24). PHQ-9 cutoff score of 10 has a sensitivity of 88% and a specificity of 88% for major depressive disorder. The PHQ-2 consists of the first two items of the PHQ-9 about the frequency of depressed mood and anhedonia over the past 2 weeks. In this study, we used the PHQ-2 cutoff score of two to enhance the sensitivity of 92.7% and the specificity of 73.7% with major depressive disorder (24).

GAD-7 (Generalized Anxiety Disorder, seven questions) is a screening tool for anxiety with recommended cutoff score is 10 or greater. The responders rate the frequency of each anxiety symptom on a four-point scale ranging from 0 (not at all) to 3 (almost every day) within 2 weeks. A cut-off of 10 has been identified as the optimal point for sensitivity (89%) and specificity (82%) (25). The Q-LES-Q-SF (Quality of Life Enjoyment and Satisfaction Questionnaire Short Form) assesses the degree of enjoyment and satisfaction in daily functioning with the total score is derived from 14 items, maximum score is 70 with higher scores indicating greater life satisfaction and enjoyment. The Vietnamese versions of GAD-7 and Q-LES-Q-SF have been translated and used most widely in clinical settings (26, 27).

### Data analysis

The quantitative data at baseline and at 3-month follow-up was entered by EpiData v3.1 software and analyzed by Stata 15.-software, determining the frequency (*n*), prevalence (%) with statistical significance *p* < 0.05, 95% confidence interval. Boxplot histograms were used to estimate the median, interquartile range. To compare PHQ-9 scores at different time points, we used analysis of variance test. Wilcoxon Signed Ranks test was used to compare PHQ-9 score, GAD-7 score, and Q-LES-Q-SF score before and after intervention. To compare the difference in the severity of depression (the PHQ-9 score) between the two times, we used the paired McNemar test. We used Generalized estimating equations regression to determine the factors associated with the outcome.

### Study ethics

The study is approved by the Institutional Review Board of Hanoi Medical University with the ethical approval No. 313/GCN-HDDDNCYSH-DHYHN December 29th, 2020.

## Results

**Figure 2:**
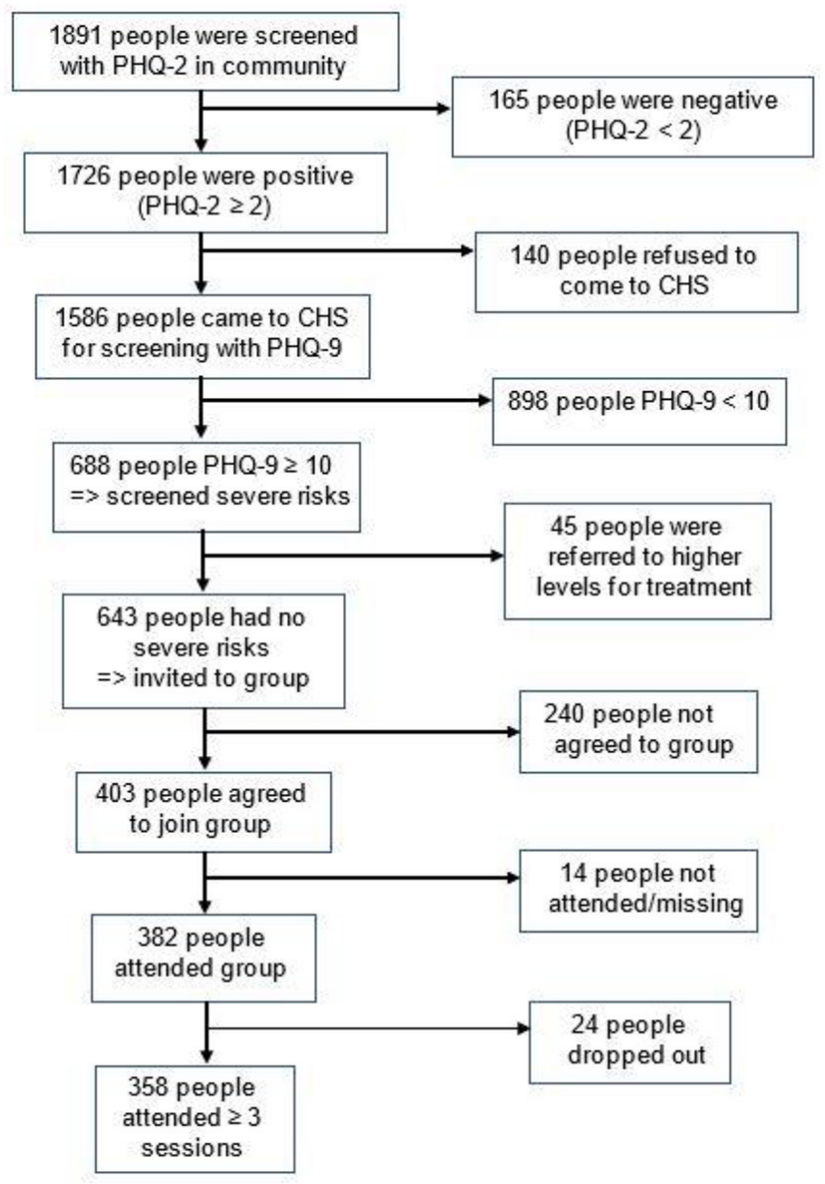
The implementation of Stepped Care Model for depression in Thai Nguyen community.

For 3 months, the program had the voluntary participation of 20 CHS staff, and 50 health collaborators/ village health workers in 10 communes. All collaborators were provided with communication leaflets for at-home screening and referral to the CHS. At the weekly meetings in the CHS, 2 CHS staff together provided the group psychotherapy and entertaining activities for 14–20 people for 90 min under the supervision of one psychiatrist. Figure 2 shows that the Stepped Care Model for depression reached to nearly 1,900 individuals in the community. The PHQ-2 positive rate was 91.3% in the community. Among those who were positive with PHQ-2 and came to CHS, 43.4% had the PHQ-9 score ≥10. The prevalence of agreement to participate in group intervention was 62.7%, in which 94.8% attended and 6.3% dropped out (i.e., attending less than three sessions).

**Figure 3:**
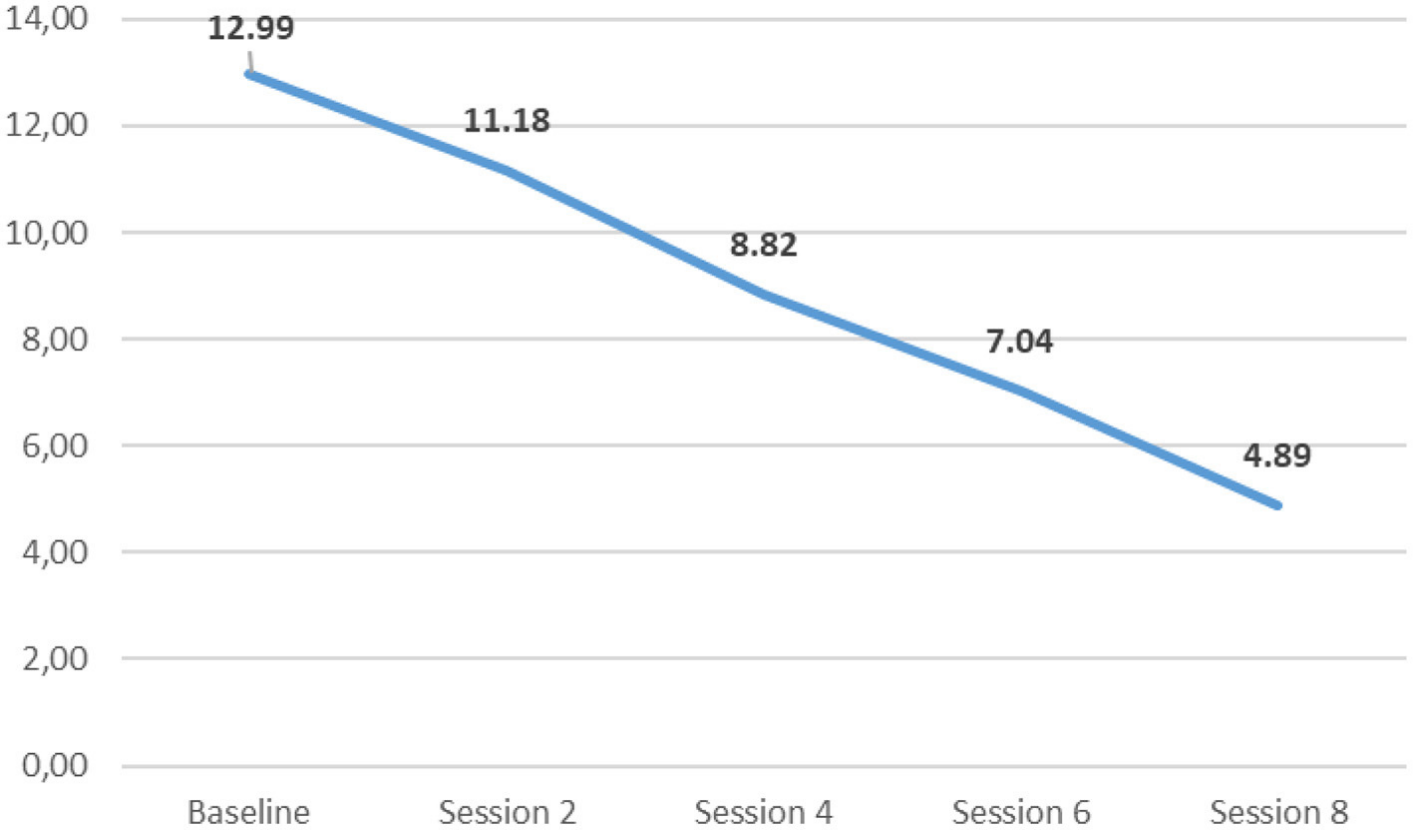
The change of depression with the PHQ-9 during group intervention.

Regarding 359 participants who met the study criteria and attended the group intervention, the majority were female, married, graduated from secondary school, and aged 55 average (Table 1). The group-based psychotherapy was conducted in 20 groups, each group had from 14 to 20 participants. Each weekly group meeting lasted ~90 min, including pleasurable activities and team-building activities to enhance the group's unity and energy. Figure 3 illustrates the significant drop of the PHQ-9 score during eight sessions of group psychotherapy.

**Table 1:** The demographic characteristics of the study subjects (*N* = 359).

**Variables**	***n***	**%**
Gender		
Male	23	6.4
Female	336	93.6
Age (Mean ± SD)	55.1 ± 7.6	
Educational level		
Primary school	25	6.9
Secondary school	144	40.2
High school	133	37.0
College and above	57	15.9
Marital status		
Single	12	3.3
Married	253	70.5
Divorced	24	6.7
Widow	70	19.5

**Figure 4:**
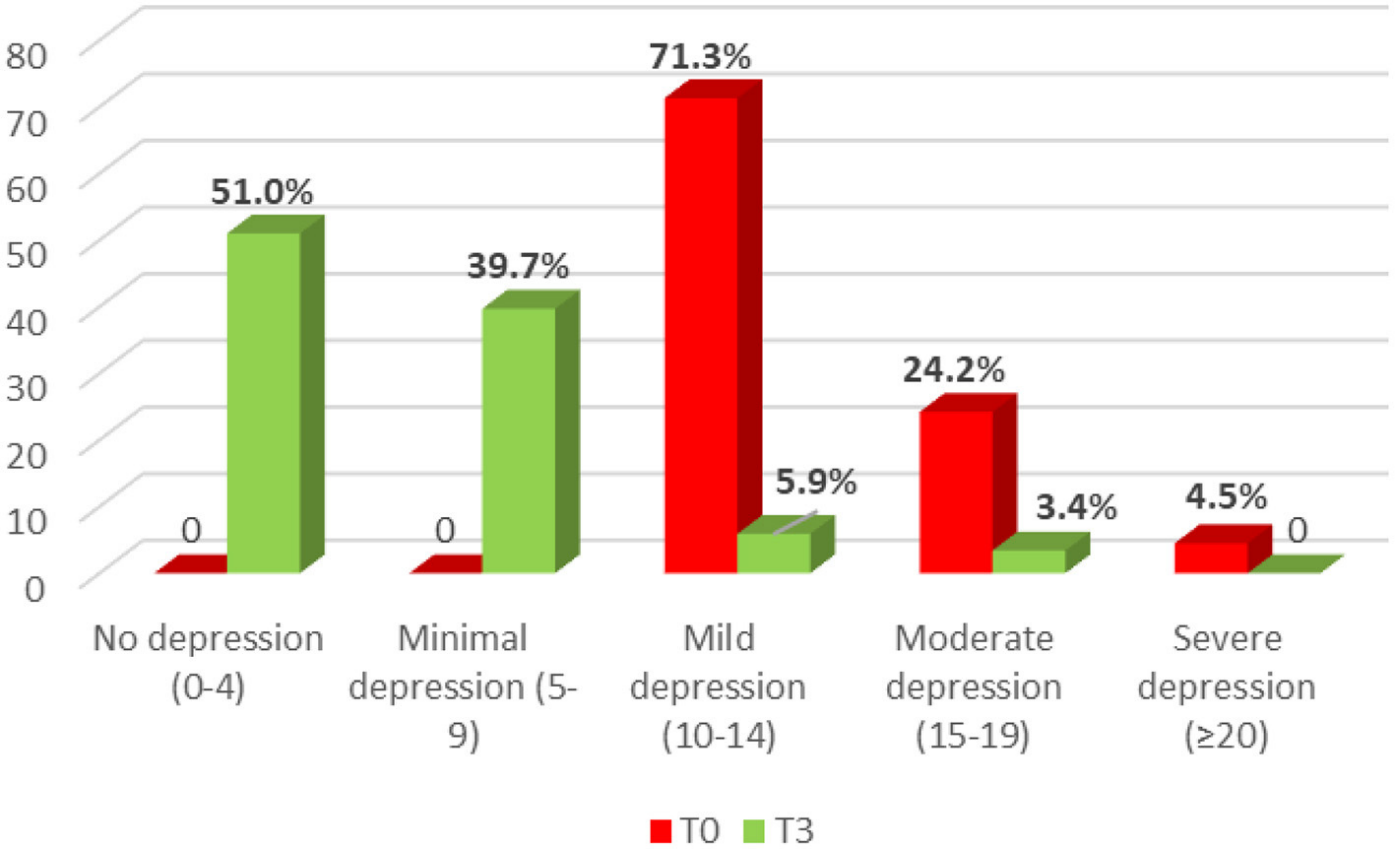
The change in depression severity based on PHQ-9 (*N* = 359).

Follow up the study group after 3 months of intervention, we noticed a significant change in depression level according to the PHQ-9 (Figure 4). It can be seen that at baseline, most of the participants had mild to moderate depression, however, after group psychotherapy, there was only <10% of individuals remained in this group. The prevalence of severe depression decreased from 4.5% to zero after 3 months. In other words, nearly 90% of patients got out of depression after group intervention. The difference in depression severity between groups was statistically significant (*p* < 0.001).

**Figure 5:**
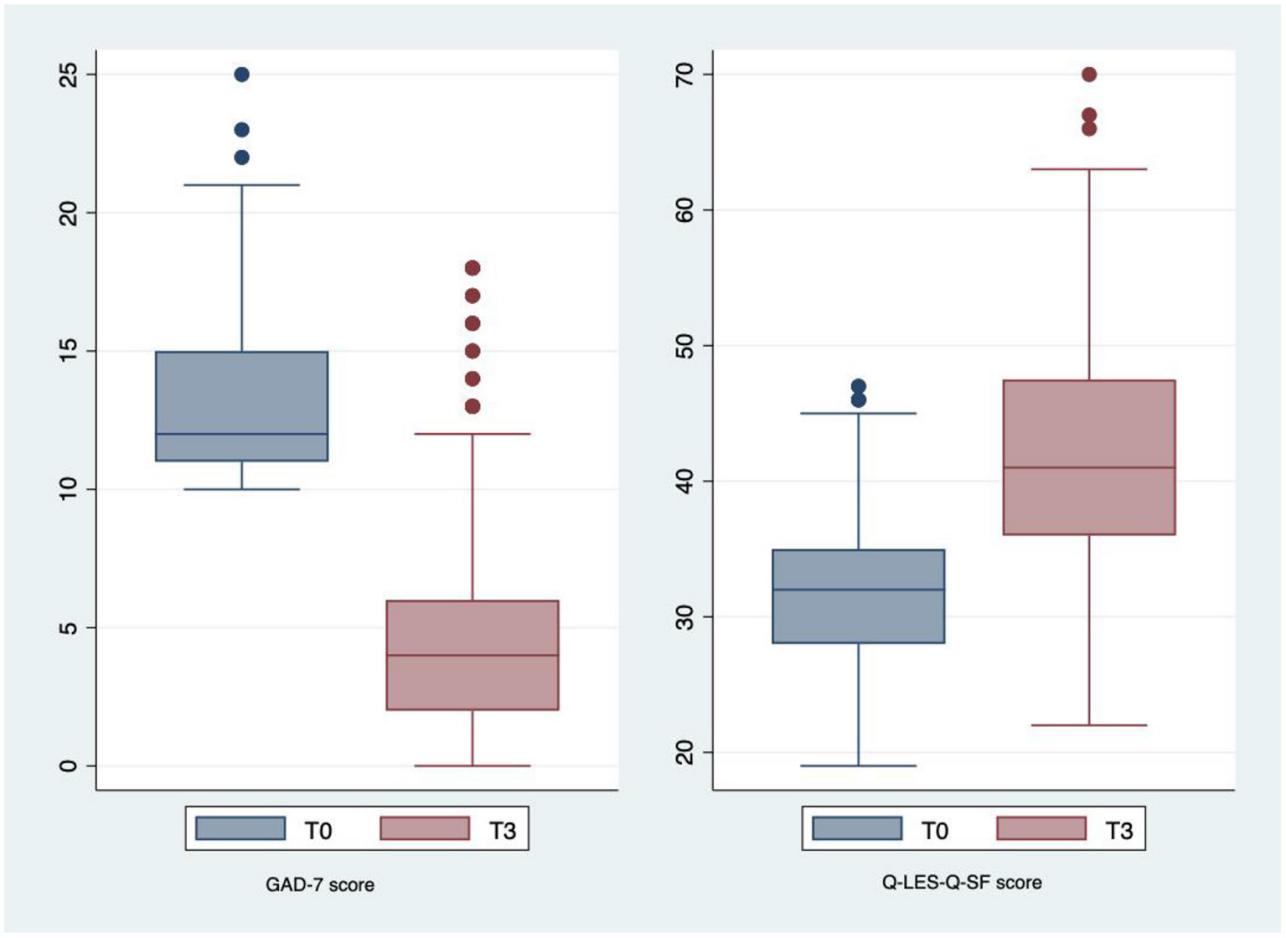
The change in anxiety level and life satisfaction in study subjects.

Figure 5 illustrates the anxiety level fell remarkably from the mild level (ranging from 5–10 scores) to the normal level (<5 scores) according to the GAD-7. Moreover,Wilcoxon Signed Ranks test showed that the pre-test median GAD-7 scores (9; IQR: 6–12) were significantly higher than the post-test median GAD-7 scores (3; IQR: 1-5) (*p* < 0.001). The median life satisfaction scores were significantly lower in the 3-month follow-up group when compared with the baseline group (T0 group: 32, IQR: 28–35; T3 group: 41, IQR: 36–47.5; *p* < 0.001). In other words, such interventions not only improved depression but also mental health and the quality of life in general.

Table 2 has shown that the results of the multivariable regression using generalized estimating equations model. Factors related to PHQ-9 score at 3 months of follow-up include: high anxiety score (*b* = 0.385, *p* < 0.001), older age (*b* = 0.045, *p* < 0.05), divorce status (*b* = 1.656, *p* < 0.05), low quality of life satisfaction score (*b* = −0.224, *p* < 0.001).

**Table 2:** Factors related to depression before and after intervention.

**Variables**	**Coef**.	**95% CI**
GAD-7 score	0.385***	0.318, 0.453
Q-LES-Q-SF score	−0.224***	−0.263, −0.185
Age	0.046***	0.015, 0.077
**Marital status**		
Single		
Married	1.29*	−0.003, 2.584
Divorced	1.656**	0.11, 3.203
Widow	1.334*	−0.016, 2.684
**Educational level**		
Primary school		
Secondary school	−0.844*	−1.792, 0.104
High school	−0.746	−1.699, 0.207
College and above	−0.202	−1.256, 0.852
**Gender**		
Female	0.037	−0.9, 0.975

^***^ p <0.01, ^**^ p <0.05, ^*^ p <0.1.

## Discussion

The Stepped Care Model for depression is a community-based approach to increase access to depression care and titrate the provision of treatment levels to need. Its core principles of delivering low-burden treatments first, followed by careful stepping up to more intensive treatment, leading to considerable implementation diversity in different context (13). In adapted Stepped Care Model for depression in Vietnam, commune and district levels (primary care) provide treatments for mild and moderate depression, and provincial level (specialty care) provide treatment for the severe cases while local collaborators provide public awareness-raising, simple psychoeducation, and detect the high-risk patients (i.e., drug abuse, suicide, psychosis, bipolar) (7).

In a previous study using simple medication and individual behavior activation therapy for mild-moderate depression, nearly 40,000 people were screened for depression with the PHQ-2, recorded 4,380 screening positive (PHQ-2 ≥3) and 2,541 people were referred to the CHS, of which 914 people were diagnosed with depression, 92% accepted treatment and 73% have completed the program. The higher positive rate after community screening in our study can be explained by the first modification of our model that focused on high-risk individuals in the community with the support of local health collaborators/village health worker. The mobilized local collaborators reaching the high-risk people at their own houses can increase access rates, create the a sense of security, and reduce resistance. Besides, the collaborators' deep understanding of particular customs and sociocultural conditions is important to facilitate the screening process, invitation, and communication to raise the awareness of the community. Another reason is that our model used the lower PHQ-2 cutoff score of 2 instead of 3 to increase the sensitivity of detecting depression in the community, leading to more positive subjects (24).

In addition, our study witnessed the lower acceptance prevalence of joining group intervention but the higher figure of completion. In the MANAS program in India, there was only 61% of patients completed treatment (11). Our findings suggest that the group interventions contribute to enhancing treatment adherence and group retention, despite the longer duration of group activities. This difference can be explained by the cultural adaptations made for Vietnamese society - increasing pleasurable group activities. These funny moments were helpful to reduce depression, improve group bonding and motivation, especially in a collectivistic society such as Vietnam. It is one of the important positive impacts of a group-based intervention that can be useful for depressive people with a high degree of isolation and less social connection.

As for the effectiveness of group-based psychotherapy, the figure showed that the PHQ-9 scores significantly decreased during eight group sessions. After 3 months, the majority of participants overcame the depression and there was no more severe case. These findings suggest that such group behavioral activation therapy helps to reduce the severity of depression in general. It is consistent with the research of the Stepped Care Model for depression in the UK with the recovery rate being 40 to 46%, and 55.4% of treated patients met reliable and clinically significant change for depression (28). The study about the behavioral activation integrating into primary health care revealed the noteworthy decreased in the PHQ-9 score after 12 months follow-up (29). In our study, the improvement of depression can be clarified by the combination of behavioral activation therapy with psychoeducation and problem-solving skills training, which are proved to be effective with depression (5, 30). Moreover, this study reveals that the group intervention not only reduces the depression level of participants but also their anxiety and quality of life after 3 months. It is in line with the research of Izquierdo showing that the community coalition approach was more effective than individual assistance at improving mental health-related quality of life at 6 months follow up (20). Similarly, the MANAS trial also proved that the effectiveness of an intervention led by trained non-specialists in primary care to improve depression and anxiety of the community at 6 months (11). The RCT of behavioral activation group therapy in patients with depression notified the less rate of drop out and better outcome in quality of life (31). It can be explained by the potential sustainable benefits of group-based behavioral activation in the increase of health habits and routines in social and daily life to recover from depression (32). That is to say, the group psychotherapy contribute to mitigating depression, and raise both the adaptive functioning and quality of life as well.

In general, prior studies on the effectiveness of this model are controversial, depending on the context and evaluation criteria. The implementation of stepped care with psychological therapies in British routine practice care for depression during 2-year follow-up showed the significant improvement in the PHQ-9 (28). It is in line with the systematic review of stepped care treatments for depression demonstrated a moderate effect with significant 6 month effect size compared to usual care (Cohen's d was 0.34; 95% confidence interval 0.20 to 0.48) (14). In contrast, the RCT in Netherlands showed the reduction of depressive symptoms in stepped care group but no difference compared to care as usual (33). However, in these western and developed countries, the care-as-usual groups usually have good access to standard treatment due to good health insurance and high awareness, which is difficult to achieve in general population of LMICs. Our study is one of the first implementation of stepped care model in the context of Vietnam with low resources, high social stigma to mental problems, and limited access to depression care. The research of stepped care intervention combined with enhanced usual care in Nigeria with similar situations presented the same effect as the enhanced usual care alone (34). However, the usual care in this study consists of many standardized care for depression such as psychoeducation, simple psychotherapy and social enhancement with proven effectiveness for depression management in the community. In fact, the concept of “standard care” or “care as usual” is very vague in many areas of Vietnam where the majority of depressed people are unrecognized and treated, while the health insurance coverage is low especially for mental problems. Therefore, any positive preliminary results of stepped care treatment for people with depressive symptoms in the community is promising to broaden on a larger scale.

During the implementation period, the intervention model has reached 1,891 people in the community who received psychoeducation on depression and primary screening with the PHQ-2 by 50 community collaborators. In 2 months, 358 people with depressive symptoms received local psychological support under the coordination of 20 commune health workers and 5 provincial psychiatrists. In general, after this study, the capacity of health workers from community level to provincial level was built to effectively deliver community-based depression care services including screening, psychoeducation, group-based psychotherapy, and appropriate referral. Besides, patients received community-based treatments that improve their mood and problem-solving skills, especially to better address the stressors related to their everyday life to improve their quality of life and functioning. In larger scale, the project helps to connect the primary health care system with the central health care system, helping to reduce the overload for provincial mental hospitals and enhance the social connection. Through communication and word-of-mouth activities in the community, the project's effectiveness helps to reduce social stigma toward mental illness, increase citizen's awareness of depression and contribute to policy advocacy for mental health. In the process of implementing the model, the research team has received the support and facilitation of the local government and the provincial mental hospital, promising to bring long-term and sustainable investments in mental health. General community in Vietnam. The long-term sustainability and feasibility of the intervention model is expected to be further evaluated by qualitative and quantitative research after 12 months of the intervention.

### Study limitation

Despite the significant model's acceptance and effectiveness, our study still has some limitations. Firstly, this is the first intervention study applying Stepped Care model in the Vietnamese community with group-based behavioral activation therapy for long-term evaluation, however, we did not have enough resources to recruit control group. For this limitation, the effectiveness of the model is not fully evaluated and certainly, which would limit the ability to draw scientifically meaningful conclusions from this study. Second, this adapted Stepped Care Model for depression is just applied for practical implementation in papers and face-to-face interaction. This may be more difficult and less feasible during the COVID-19 pandemic that requires remote interaction and electronic contact. Thirdly, the sampling attempted to diversify the population by rolling out in 10 different communes, but the implementation of the adapted model preliminary focused on high-risk groups of depression in the community. Therefore, the study results represent better for vulnerable populations in Vietnam. For these reasons, there is a need for more intervention studies with representative samples and control group for long-term follow-up to fully evaluate the acceptance and the effectiveness of this model in the community. Nevertheless, the modifications to the Stepped Care Model focusing on group behavior activation therapy described in this paper are the first step for further studies on depression management in Vietnam as well as other LMICs in the future.

## Conclusions

Depression is the leading mental health burden worldwide, and the Stepped Care Model is proved to be an effective approach for depression management in low-resource communities. This study focuses on the adaptation and implementation of this model in the Vietnamese context and lessons learned. These preliminary results imply that the adapted Stepped Care Model should be considered as a suitable and promising method to control the depression in the Vietnamese community with limited resources and high cost-efficiency. Further studies should be implemented to examine the long-term effectiveness of this model and group psychotherapy to promote public mental well-being.

## Data availability statement

The raw data supporting the conclusions of this article will be made available by the authors, without undue reservation.

## Ethics statement

The studies involving human participants were reviewed and approved by the Institutional Review Board of Hanoi Medical University. The patients/participants provided their written informed consent to participate in this study.

## Author contributions

MD and HT designed the study. MD and TN collected and analyzed the data. MD, TN, and HT interpreted the data and drafted the manuscript. TN and HT reviewed the data analysis and manuscript. All authors approved the final version for publication.

## Conflict of interest

The authors declare that the research was conducted in the absence of any commercial or financial relationships that could be construed as a potential conflict of interest.

## Publisher's note

All claims expressed in this article are solely those of the authors and do not necessarily represent those of their affiliated organizations, or those of the publisher, the editors and the reviewers. Any product that may be evaluated in this article, or claim that may be made by its manufacturer, is not guaranteed or endorsed by the publisher.
